# Effects of Fructose and Oligofructose Addition on Milk Fermentation Using Novel *Lactobacillus* Cultures to Obtain High-Quality Yogurt-like Products

**DOI:** 10.3390/molecules26195730

**Published:** 2021-09-22

**Authors:** Dorota Zielińska, Katarzyna Marciniak-Lukasiak, Marcelina Karbowiak, Piotr Lukasiak

**Affiliations:** 1 Department of Food Gastronomy and Food Hygiene, Institute of Human Nutrition Sciences, Warsaw University of Life Sciences (WULS-SGGW), Nowoursynowska 159c, 02-776 Warsaw, Poland; marcelina.karbowiak@gmail.com; 2 Department of Food Technology and Assessment, Division of Fat and Oils and Food Concentrates Technology, Institute of Food Sciences, Warsaw University of Life Sciences (WULS-SGGW), Nowoursynowska 159c, 02-776 Warsaw, Poland; 3 Faculty of Computing and Telecommunications, Institute of Computing Science, Poznan University of Technology, Piotrowo 2, 60-965 Poznan, Poland; piotr.lukasiak@put.poznan.pl; 4 Laboratory of Bioinformatics, Institute of Biochemistry Polish Academy of Sciences, Noskowskiego 12/14, 61-704 Poznan, Poland

**Keywords:** *Lactobacillus*, oligofructose, fructose, milk fermentation, texture, sensory properties

## Abstract

The incorporation of prebiotics in fermented milk products is one of the best ways to promote health benefits while improving their sensory characteristics at the same time. The aim of this study was to evaluate the effects of the addition of fructose and oligofructose (1% and 2%) on the physicochemical, rheological, sensory, and microbiological quality attributes of fermented milk products inoculated with indigenous probiotic starter cultures of *Lactobacillus* isolated from Polish traditional fermented foods. The samples were evaluated during 35 days of refrigerated storage. The oligofructose and fructose caused increases in the populations of bacteria in comparison to the control fermented milk products without the addition of saccharides. The degrees of acidification in different fermented milk samples, as well as their viscosity, firmness, syneresis, and color attributes, changed during storage. The highest overall sensory quality levels were observed for the samples supplemented with *L. brevis* B1 and oligofructose. This study is the first attempt to compare the influences of different sugar sources on the physicochemical, rheological, sensory, and microbiological quality attributes of fermented milk products.

## 1. Introduction

Lactic acid bacteria (LAB) play a multifunctional role in food, agricultural, and clinical applications. Using LAB in the food fermentation process is one of the ancient food preservation techniques. Fermented milk products, such as yogurt and cheese, appeared in the human diet about 8000–10,000 years ago. Until the 20th century, food fermentation remained an unregulated process. The discovery and characterization of LAB changed the perception of food fermentation [1]. The prerequisite for the use of LAB strains in dairy starter cultures is their qualified presumption of safety (QPS) status as recommended by the EFSA (2018) [2]. Starters are microbial cultures used to promote and facilitate the fermentation of dairy products. The primary function of starters is the production of lactic acid from lactose. Other functions may include enhancing the flavor and aroma, alcohol production, proteolytic and lipolytic activities, and the inhibition of undesirable organisms [3]. Microbial starter cultures play a crucial role in the formation of bioactive components, which impart antioxidant, antihypertensive, antidiabetic, and antiallergic potential to dairy products [4].

Among the many microorganisms that produce lactic acid through fermentation, only a few are considered probiotic species. According to the International Scientific Association for Probiotics and Prebiotics (2014), “live microorganisms that when administered in adequate amounts confer a health benefit to the host” are considered as probiotics [5]. In order for probiotics to be beneficial for the host, firstly they should be able to survive gastric transit and reach the small intestine in sufficient numbers. The incorporation of probiotics into dairy foods may aid in tolerating gastrointestinal conditions, as the buffering action of milk and milk fat may protect probiotics by reducing their direct exposure to harsh conditions [6]. In recent years, fermented dairy foods have been considered some of the most common and traditional vehicles for delivering probiotics to humans. Probiotics are increasingly used as starter cultures or in combination with traditional cultures. The criteria used to assess the suitability of a strain to be used as a starter in food products or as a functional adjunct are as follows: the growth kinetics and total production of acids; pH changes; losses of substances with high nutritional value; the type of metabolism; the ability to impart desirable sensory properties [7].

From a biological point of view, fermented milk products are characterized by the accumulation of microbial metabolic compounds, such as lactic acid or ethyl alcohol, which are collectively called flavor substances and which contribute to the overall preservative action [8]. The lactic acid is responsible for the development of the characteristic body and texture attributes of fermented milk products, contributes to the overall flavor of the products, and enhances their shelf life. Bacteriocins, which are synthetized by lactic acid bacteria, can also serve as natural preservatives. Diacetyl, acetaldehyde, and acetic acid contribute to the flavor and aroma of the final products. During fermentation and storage, several bioactive peptides can also be released by proteolytic cultures, such as dipeptidases, tripeptidases, aminopeptidases, and endopeptidases, which are beneficial for human health [7].

It has been shown that traditional fermented foods are good sources of isolated bacteria with probiotic properties, which are comparable to strains isolated from the human digestive tract [9]. Obtaining new probiotic strains isolated mostly from artisanal, spontaneously fermented food products enables the use of new starter cultures and will allow the production of a wide range of health-promoting foods [10]. The selection of an appropriate starter culture type, such as a traditional culture, probiotic adjunct culture, or other combinations, is of great importance in the manufacturing of fermented milk products [11]. Novel trends in fermented dairy technology contribute to the creation of various products with high nutritive and lower energy values possessing functional properties. Dairy-based matrices are suitable for the proliferation of probiotics due to supplying rich sources of carbon and essential amino acids as a consequence of lactose hydrolyzation and the presence of proteolytic systems involved in casein utilization [12].

Milk can also be supplemented with different prebiotic compounds as growth factors and growth promoters to enhance the survival of probiotics [13]. Prebiotics are selectively fermented ingredients that cause specific changes in the composition or activity of the gastrointestinal microbiota, thereby conferring health benefits to the host [14]. The incorporation of prebiotics into fermented milk products is one of the ways to promote health benefits while improving sensory characteristics at the same time [15]. Furthermore, prebiotic sources are also used to manage water migration and to enhance a food product’s taste, mouthfeel, texture, and shelf life without significantly altering its specific application characteristics [16]. A product that contains both probiotic and prebiotic agents and that has numerous positive effects on the health and intestinal microbiota of the host can be defined as synbiotic [17].

To date, the most commonly used sweeteners in the dairy industry are sucrose, corn syrup, and honey. Based on this background, inulin-like fructans are important food ingredients that should be further explored for the production of new functional, low-calorie dairy products. Oligofructose is a naturally occurring carbohydrate that contains soluble dietary fiber. It is also a linear nondigestible oligosaccharide of β-(2-1)-linked fructose residues with a terminal glucose residue unit, which is obtained from the partial enzymatic hydrolysis of inulin [18]. It selectively stimulates the growth of species of *Bifidobacterium* in vitro, a genus of bacteria that is considered beneficial to health [19] and is, therefore, called a prebiotic.

Although various studies have evaluated the impacts of oligofructose addition at concentrations ranging from 0.1% to 5% on the growth of several species of *Lactobacillus* in different food matrices, as well as their physicochemical compositions, the results seem inconsistent [20,21,22,23,24,25,26,27,28]. Although in most studies the addition of prebiotics positively affected the survivability of bacteria, the ability to ferment prebiotics is strain-specific for *Lactobacillus.* Additionally, the acidification rates, as well as the influence on the textural and sensory properties, differ depending on the probiotic or prebiotic agent that is added. Moreover, only a few studies have presented the effects of fructose or fructose sources on *Lactobacillus* survival and the overall quality of fermented milk [29,30,31]. Importantly, there is a lack of studies that comprehensively compare the influence of sugar addition on the physicochemical, rheological, sensory, and microbiological quality of fermented milk products during cold storage; therefore, the aim of this study was to evaluate the effects of the fructose and oligofructose addition on the characteristics of fermented milk products inoculated with indigenous probiotic starter cultures of *Lactobacillus* isolated from traditional Polish fermented foods.

## 2. Results

### 2.1. Lactobacillus Count Changes in Fermented Milk Samples during Storage

**Figure 1:**
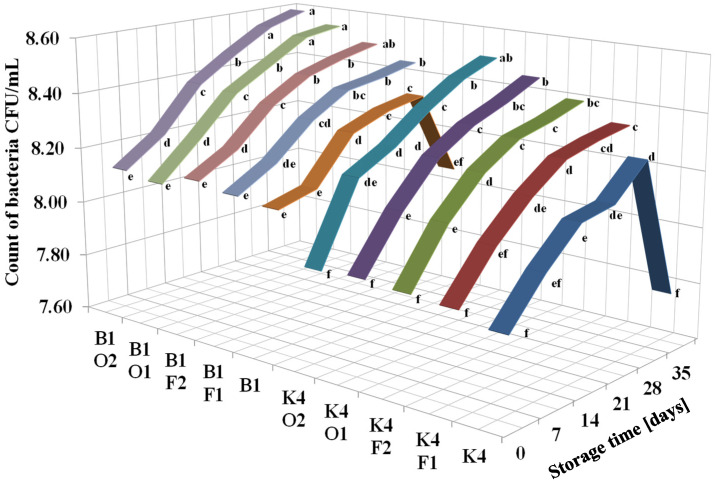
Changes in the *Lactobacillus* counts in fermented milk samples during storage. The samples were coded according to Materials and methods section, whereby values marked by different letters represent statistically significant differences between variables (storage time and strains) (*p* < 0.05) (*n* = 3).

In the present study, two *Lactobacillus* strains, namely *L. brevis* B1 and *L. johnsonii* K4, were used in combination with different carbon sources, namely fructose (F) and oligofructose (O), at concentrations of 1% and 2%. It was found that directly after fermentation, the *Lactobacillus* counts in all K4 samples (7.88–7.94 CFU/mL) were significantly lower than in B1 samples (8.10–8.14 CFU/mL) (Figure 1).

The bacterial counts in all fermented milk samples with the addition of F and O gradually increased over 35 days of refrigeration in the storage test. Only in control samples without the addition of sugar (K4 and B1) did the levels of *Lactobacillus* significantly decrease between day 28 and day 35 of storage. The most effective growth stimulator was the addition of 2% oligofructose; however, the addition of 1% oligofructose and 2% fructose resulted in most cases in similar stimulation effects to that observed for 2% oligofructose.

### 2.2. The pH Changes of Fermented Milk Samples during Storage

The pH values of the fermented milk samples changed significantly during storage. In samples fermented with *L. johnsonii* K4, the average pH value was approximately 4.8 directly after fermentation, in contrast to samples fermented with *L. brevis* B1, for which the average pH value at the beginning was approximately 4.3 (Table 1). 

During refrigeration storage, the pH values decreased in all samples. This phenomenon was most apparent between 7 and 21 days. The reductions in pH values were more significant in K4 samples than in B1 samples. Between 21 and 35 days of storage, the pH values remained stable in almost all samples, except K4 and K4 F1, in which stabilization was observed after 28 days of storage. The addition of saccharides also affected the acidification kinetics. Acidification was more dynamic when oligofructose was added in comparison to fructose, regardless of the initial sugar concentration (1% or 2%). The initial pH value at the beginning of inoculation was at the same level of approximately 6.70 ± 0.10. After 48 h of fermentation, the pH values of samples with added oligofructose dropped to 4.31–4.32 for B1 samples and 4.68–4.69 for K4 samples, whereas in samples with added fructose the values were more varied, in the range of 4.31–4.38 for B1 samples and 4.70–4.83 for K4 samples, depending on the fructose concentration.

### 2.3. Viscosity, Firmness, Syneresis, and Color Changes in Fermented Milk Samples during Storage

The viscosity levels (Table 2) of all milk samples fermented with *L. johnsonii* K4 were significantly lower than in samples fermented with *L. brevis* B1, and these changed during storage. In all samples, the viscosity values increased significantly (*p* < 0.05) between day 0 and day 7 of storage, with the highest values (>100 mPa·s) obtained in samples with added oligofructose, namely K4 O2, B1 O1, and B1 O2. Between day 7 and day 21, significant decreases (*p* < 0.05) in viscosity were observed in almost all samples except B1, B1 F1, and B1 F2. At day 35 of the storage test, the viscosity values for the fermented milk samples were comparable or slightly higher than the initial values.

The changes in firmness values (Table 3) were similar in all fermented milk samples, regardless of the addition of the *Lactobacillus* strain. Between day 0 and day 7 of storage, the firmness values increased significantly (*p* < 0.05) in almost all samples, except K4 F1, K4 O2, and B1 F1. Next, between day 7 and day 21 or day 35 of storage, the firmness values decreased significantly (*p* < 0.05) to values lower or similar to the initial values.

**Figure 2:**
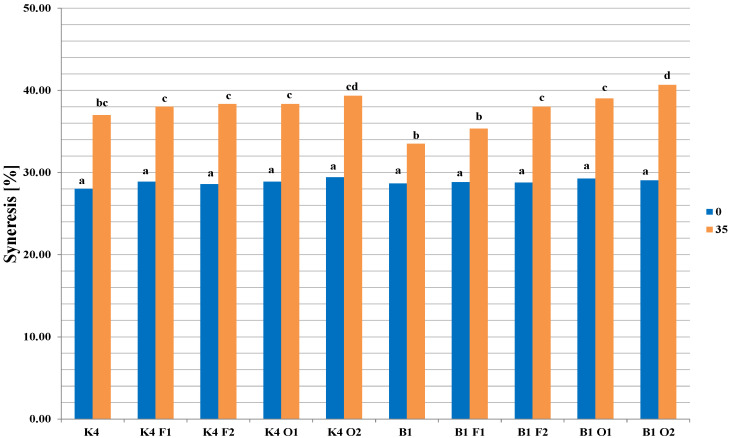
Syneresis in fermented milk samples at 0 and 35 days of storage. The samples are coded according to Materials and methods section, whereby values denoted by different letters represent statistically significant differences between variables (storage time and strains) (*p* < 0.05) (*n* = 3).

The results highlighting the degree of syneresis in the fresh samples (day 0) and in samples after 35 days of storage are shown in Figure 2. It was found that in fresh samples, the levels of syneresis were very similar (approx. 28%–29%), regardless of which strain was added, although the values increased significantly (*p* < 0.05) during storage (33.5%–40.7%). The biggest syneresis increase was observed in samples with 2% oligofructose addition, while the smallest increase was in samples without saccharides (K4 and B1 samples) or with 1% fructose addition (B1 and F1 samples).

The results for the color quality parameters of the fermented milk samples are shown in Table 4. The values of parameter L*, which indicates brightness, were in the range of 83.06–92.48 and increased significantly (*p* < 0.05) up to day 21 of storage. The L* values were slightly higher for K4 samples. The values of the measured color parameters, such as the psychometric tone (a*), were negative up to day 35 of storage for both groups of fermented milk samples. Moreover, a* values decreased during storage up to 21 day. The calculated values for psychometric chroma (b*) for the samples were positive throughout the entire storage period, indicating the presence of yellow components such as the yellowish-green-colored riboflavin (vitamin B_2_), β-carotene, and vitamin A molecules [32]. The b* values increased during storage in all samples up to day 21. Between day 21 and day 35 of storage, L* and b* parameters decreased, whereas a* values significantly increased in almost all samples. To ensure a comprehensive analysis of the effects of the different probiotic cultures and saccharide addition on the color of the fermented milk products, the total color difference parameter was calculated (ΔE). It was found that the differences in color between samples with and without the addition of probiotics and prebiotics were unnoticeable, even for the inexperienced observer (ΔE < 5). 

### 2.4. Sensory Characteristic

**Figure 3:**
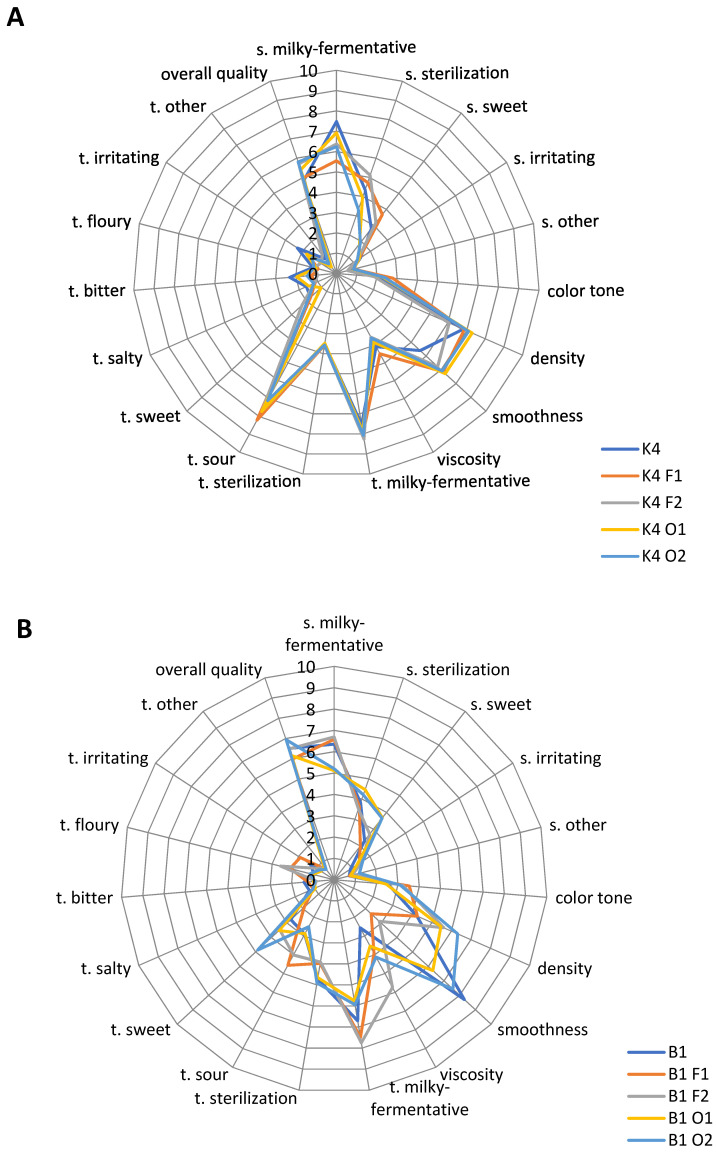
Sensory profiles of milk samples fermented with *L. johnsonii* K4 (**A**) and *L. brevis* B1 (**B**). Samples codes according to Materials and methods section; (*n* = 3).

The results of the profiling analysis of the ten fermented milk samples are presented in Figure 3A,B. The results showed that the samples were characterized by different sensory profiles.

Milk samples fermented with *L. johnsonii* K4 (Figure 3A) were characterized by an intense milky-fermentative smell (5.5–7.5 c.u.), a typical sterilization smell (3.3–5.1 c.u.), and a less intense sweet smell (1.9–3.7 c.u.). The visual attributes were similar in all samples. The color tone was assessed as light, while the samples were quite dense (6.1–7.2 c.u.) and smooth (5.6–7.2 c.u.). The viscosity was characterized as low (3.6–4.5 c.u.). The taste was dominated by a milky-fermentative (7.5–8.3 c.u.) and sour (7.5–8.2 c.u.). A less intense sour taste was observed in the control sample. The samples showed low-intensity sweet (1.2–1.7), bitter (0.9–2.3 c.u.), and salty (1.1–1.5 c.u.) taste attributes. Other negatively correlated tastes (floury, irritating, other) were of low intensity (<2 c.u.). The overall quality, which depended on all of the characteristics perceived in the fermented milk samples, varied across the samples. The highest overall quality was observed for the K4 O2 sample (5.8 c.u.), whereas control sample K4 showed the lowest quality (4.8 c.u.).

Milk samples fermented with *L. brevis* B1 (Figure 3B) were characterized by a typical milky-fermentative smell (5.1–6.7 c.u.) and a sterilization smell (3.5–4.5 c.u.). The sweet smell was less intense (2.0–3.7 c.u.) and the negative smell notes (irritating and other) were also less intense (<2 c.u.). The color intensity was low (2.4–3.5 c.u.), while other appearance characteristics differed depending on the saccharide that was added. The density was highest in the B1 O2 sample (6.3 c.u.), while the lowest density values were observed in the B1 control sample and B1 F1 sample (4.2 and 4.3 c.u., respectively). The smoothness was the highest in the B1 control sample (8.3 c.u.), followed by the B1 O2 sample (7.6 c.u.). The intensity of the viscosity varied (2.6–5.7 c.u.) depending on the sugar that was added. The taste of the B1 samples was more harmonious than in K4 samples. The intensity of the typical milky-fermented taste was high (5.7–7.7 c.u.), as well as the sterile taste (3.9–4.9 c.u.). The sour taste was less intense (2.5–4.5 c.u.), similarly for the sweet taste (1.9–4.8 c.u.), depending on the saccharide that was added. Other tastes (salty, bitter, floury, irritating, and other) were less intense (<2 c.u.). The overall quality of B1 fermented milk samples was rather high (5.9–6.9 c.u.), which indicated that this strain possesses favorable features.

**Figure 4:**
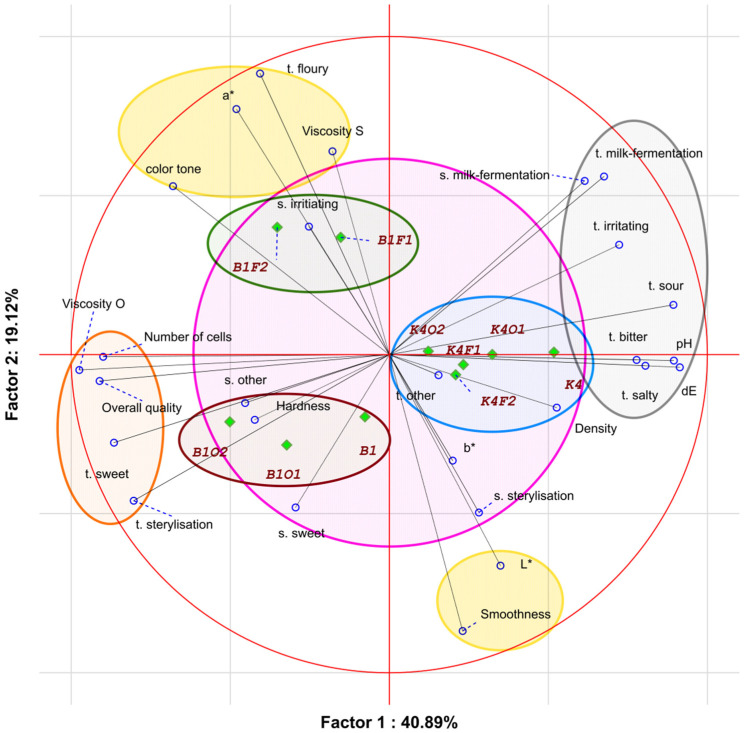
PCA analysis results shown via a projection of the variables and cases on the factor plane. The samples were coded according to Materials and methods section. The abbreviation ‘t.’ corresponds to taste, while ‘s.’ corresponds to smell; L*, a*, and b* correspond to color components.

The principal component analysis of the results (Figure 4) of the sensory profile and textural evaluation of all ten fermented milk samples showed that the variability of the samples was due to the six main components, which accounted for 95.1% of the total variability and defined the relationships between the samples and the factors defining their quality; however, to make the results more transparent, two main components were selected for further analysis.

Factor 1 accounted for 40.89% of the total variability and was related to dE, pH, organoleptic viscosity, overall quality, and taste (milky-fermentative, irritating, sour, bitter, salty, sweet), as well as the milk fermentation smell. The second component (factor 2) accounted for 19.12% of the general variability and was related to the floury taste, smoothness, sensory viscosity, and color components (L* and a*).

From the PCA image and the locations of the samples, it can be seen that the fermented milk samples differed in quality. In addition, they formed three distinct clusters. One of them consisted of samples containing *L. johnsonii* K4 culture control and other *L. johnsonii* K4 cultures, with various additions of fructose and oligofructose. The second cluster consisted of the *L. brevis* B1 culture control sample and *L. brevis* B1 culture with 1% and 2% additions of oligofructose. The last cluster consisted of the *L. brevis* B1 culture sample with added fructose. The obtained clusters clearly distinguished the samples made with different cultures. The milk samples fermented with *L. johnsonii* K4 culture were characterized by higher ΔE factor, pH, and density values. Additionally, a more intense taste was found in these samples (milky-fermentative, irritating, sour, bitter, and salty) than in samples with *L. brevis* B1.

One can notice that the sweet and sterile tastes mostly corresponded to the samples made with *L. brevis* B1. These samples, besides their sweet taste, were characterized by higher organoleptic viscosity values and higher numbers of cells. It is worth noting that samples made with *L. brevis* B1were better evaluated in terms of the overall quality. As was mentioned, samples made with *L. brevis* B1 were divided into two clusters based on the type of saccharide that was added. Samples containing oligofructose were characterized by a sweet smell, while samples containing fructose were characterized by a floury taste and higher sensory viscosity, color tone, and a* component values.

Based on the above analysis, one can conclude that samples made with *L. brevis* B1 were characterized by different factors than samples made with *L. johnsonii* K4. The addition of fructose in the amount of 1% caused an increase in sensory viscosity and the appearance of an irritating smell. Further addition of fructose up to 2% changed the color tone of the samples. The addition of oligofructose in the amount of 1% increased the hardness and caused the appearance of a sweet smell. The addition of oligofructose up to 2% caused increases in organoleptic viscosity and overall quality, as well as a more intense sweet and sterile taste.

The analysis of the sensory quality attributes of the tested samples showed that the overall quality was positively correlated with the organoleptic viscosity and taste (sweet and sterile), as well as high numbers of lactic acid bacteria. Negative correlations with overall quality were detected for pH, ΔE, and taste (milky-fermentative, sour, bitter, salty, irritating) attributes. Additionally, density and taste were correlated both with each other and with K4 samples.

## 3. Discussion

The most important factors to consider when novel probiotic adjunct cultures are applied in developing dairy products are their effects on the fermentation process and product quality and the ultimate acceptability to consumers; therefore, our study investigated whether and how the addition of different *Lactobacillus* strains affect these aspects. Moreover, two different carbon sources were tested, namely fructose and oligofructose.

Oligofructose is recommended as a prebiotic and is usually added to products at 1%–50% to ensure fiber enrichment along with additional nutritional and health benefits, such as the stimulation of probiotic bacteria [33]. The beneficial effects of oligofructose on human health are not only limited to its use as a dietary fiber (intestinal passage control, reduction of cholesterol, and decreased risk of osteoporosis by increasing mineral absorption) [34,35], but also include the benefits derived from its prebiotic nature. Oligofructose can only be digested through bacterial activity; it can alter the composition of human gut microbiota via a specific fermentation process, which results in a community predominated by bifidobacteria [36]. It also inhibits the growth of pathogens and harmful microorganisms and causes a reduction in intestinal pH [37]. Moreover, the recent interest from scientists has focused attention on the effects of the addition of oligofructose to dairy products to improve the rheological, sensory, and physicochemical attributes [20,21,38,39]. All of the above properties of oligofructose make it one of the most popular prebiotic additives in the milk fermentation industry.

In contrast, for many years fructose has been associated with negative impacts on human health; nonetheless, fructose additives are still used in dairy production, whether in the form of fruits, honey, or corn syrup. Many studies have shown increased consumption of high-fructose corn syrup, as well as total fructose, over the past 20 to 30 years, which has been linked to increases in obesity and metabolic disorders [40]. Rizkalla [41] claimed that this raises concerns regarding the short- and long-term effects of fructose in humans. On the other hand, the results presented in some studies have shown that the addition of honey has a stimulatory effect on the growth of probiotic strains, such as for *Lactobacillus casei* Lc-01 in cow and goat milk [42] or *Lactobacillus reuteri* DPC16 in manuka honey [29]. Recently, there has been a growing interest in the use of honey as an alternative natural sweetener in processed foods, owing to its positive image among consumers [43].

The results of our study showed that both saccharide sources (F and O) significantly stimulated the growth of starter cultures of *Lactobacillus* in comparison to control samples; however, the best survivability of probiotic bacteria was observed in samples made 2% and 1% oligofructose and 2% of fructose. The *Lactobacillus* counts in all samples were high (approx. 8 CFU/mL) during the 35 days of storage, which is an important factor in the probiotic food development process. Acidity changes in fermented milk beverages were also observed. The pH values of samples varied depending on the strain, as well as the saccharide source used in the study. Directly after fermentation, *Lactobacillus brevis* B1 caused the pH value to drop to 4.31–4.38, whereas *L. johnsonii* K4 caused the pH value to drop to 4.68–4.85. Lower pH values were observed in the samples containing 2% oligofructose and fructose. The pH values decreased up to day 21 of refrigeration, after which they stabilized.

The other results obtained for probiotic bacteria strains used as starter cultures together with the addition of carbohydrate compounds showed varying levels of growth on milk during the fermentation and refrigeration stages. It has been found that probiotic bacteria show poor acidification performance in milk compared with common yogurt starter cultures [44,45,46]. Oligofructose and other prebiotics can increase the survivability of bacteria. For instance, Cruz et al. [20] observed counts of 9.34, 9.56, 8.56, and 8.21 log CFU/mL for the strains *S. thermophilus* and *L. bulgaricus* with the addition of 2%, 4%, 6%, and 8% oligofructose. Similarly to our study, Fornelli et al. [21] observed significant increases in *L. paracasei* populations between the 7th and 14th day of refrigerated storage (*p* < 0.05); however, between the 14th and the 21st day of storage, significant reductions in *L. paracasei* populations (*p* < 0.05) were observed. During refrigerated storage, statistically significant reductions (*p* < 0.05) in pH value were observed after 21 days. Additionally, Oliveira et al. [23] evaluated the effects of prebiotics on the fermentation profile in a synbiotic low-fat milk and found that the mean viable counts of the probiotics were 7.8% higher than in the control. Kadrin et al. [24] found that that the optimal quantity for an added prebiotic ingredient to ensure adequate populations of *L. acidophilus* in samples over the shelf life period was 1%. Similarly, Gustaw et al. [25], when studying the addition of oligofructose to yogurt, caused an increase in the numbers of all bacteria in comparison to control yogurt obtained without the addition of prebiotics. Oligofructose at the concentration of 1% was useful for *L. acidophilus* because it kept these bacteria were stable for the entire storage time. At higher oligofructose concentrations (2 and 3%), significant decreases in all bacteria populations were observed during refrigerated storage. In turn, Aghajani et al. [26] found that the highest probiotic counts were related to a sample containing inulin in their study. Recent studies by Chand et al. [27] and Kariyawasam et al. [28] have also confirmed the above-mentioned results from other authors’ studies.

All of these results suggest that oligofructose can be used by microorganisms as a nutrient source. This effect is most likely due to the release of fructose during its partial hydrolysis and subsequent metabolization as an additional carbon and energy source [47]. Probiotic bacteria possess cell-associated glycosidases that hydrolyze oligosaccharides to form monomers of fructose, which can be fed into the phosphoketolase pathway [48]. Further, an operon involved in oligofructose utilization has been described for *L. acidophilus* NCFM [49]. It has been demonstrated that certain *Lactobacillus* strains can grow on inulin-type fructans [20,21,22,23,24,25,26,27,28,29,30,31]; however, some authors have indicated that the ability to ferment these prebiotics is strain-specific for *Lactobacillus*, in contrast with *Bifidobacterium*, in which these properties are more widespread [50]. *L. brevis* has commonly been found to be heterofermented and to employ the phosphoketolase pathway while also possessing inducible glycolytic enzymes [51]. On the other hand, *L. johnsonii* is a homofermented bacterium, which shows highly glycolytic properties [52]. Our findings prove that both strains can utilize oligofructose as a carbon source.

To the best of our knowledge, limited studies have considered the addition of fructose in the production of fermented milk beverages as compared with the addition of oligofructose; however, Saxena et al. [30] found that the addition of fructose significantly enhanced the viable counts and sugar utilization of different *L. acidophilus* strains as compared to the control. On the other hand, Hartati et al. [53] showed that adding date palm (containing glucose, fructose, and sucrose) as a sugar to yogurt beverages can optimize the growth of lactic acid bacteria. Additionally, Pangetika et al. [31] found that *Lactobacillus acidophilus* growth in medium containing 3% *D*-fructose caused a ±3% change in pH as compared to the medium containing no *D*-fructose. The decrease in pH value might be explained by bacterial activity in the organic acids produced during fermentation. In the study by Slačanac et al. [42], it was proven that the addition of acacia honey resulted in a higher number of *Lactobacillus casei* Lc-01 cells and lower pH values in fermented milk than with the addition of chestnut honey.

Our study indicated that both oligofructose and fructose are good carbon sources for the tested *Lactobacillus* bacteria strains, as both enhanced their survivability; however, the degree of acidification, as well as the viscosity, firmness, syneresis, color, and sensory properties of fermented milk samples, were changed during storage.

Based on the performed analyses, it can be concluded that *L. brevis* B1 and *L. johnsonii* K4 starter cultures had different impacts on the physicochemical properties of the product. Dairy products with the addition of the B1 strain were characterized by a compact texture, resembling the classic form of yogurt. In turn, the samples with the addition of the K4 strain were characterized by lower viscosity and increasing liquidity. The composition of the carbohydrates was also one of the basic factors influencing the sensory features, texture, and rheological properties of fermented milk samples. The 2% oligofructose addition caused significant increases in the apparent viscosity and hardness of fermented milk formulations in comparison to control products. It could be assumed that oligofructose is part of the structural network being formed during the fermentation and structuring of fermented milk products. In the case of fructose addition, a smaller improvement in terms of the rheological properties was observed for the final products. Castro et al. [38] showed that oligofructose did not show any significant influence on the technological properties (fermentation time, acidity, and syneresis index) or the population of probiotic bacteria in fermented lactic beverages. The same authors [22] in another study reported that the viscosity decreased with increasing oligofructose concentration in the manufacture of the lactic beverages. They concluded that the phenomenon observed in the study may be related to a possible plasticizing effect of oligofructose, resulting in less moisture and a reduction of the hydrodynamic volume of the protein, decreasing the viscosity. Similarly, Glibowski and Zielińska [54] substituted some of the skim milk powder for oligofructose in stirred kefir products and did not notice any changes of the rheological behavior. Regardless of the storage time, kefirs with added oligofructose required much less energy to destroy the structure. The lowest apparent viscosity was measured for kefirs with native inulin and oligofructose. Completely different results were presented by Gustaw et al. [25], who obtained higher contents of solids in their yogurt samples, which promoted greater viscosity of the final products. These observations were in agreement with our study, as well as with others [55,56]. Syneresis is undesirable in fermented milk products process of the resulting whey separation from the curd after coagulation. Syneresis can affect the quality of yogurt-like products, especially in terms of their consistency and viscosity. Whey loss measures the level of gel collapse and is an indicator of poor quality and stability. Syneresis may be spontaneous or may occur only when the gel is mechanically disrupted by cutting, agitating, or being subjected to a centrifugal force. In the present study, the levels of syneresis were rather high (28%–29%) in the initial phase, which increased significantly during storage. Mani-López et al. [44] reported >40% syneresis when probiotic cultures were applied in fermented milk formulations. Moreover, Amatayakul et al. [57] showed syneresis values >45% in yogurts fermented with exopolysaccharide-producing starter cultures. In turn, syneresis values ranging from 23 to 36 ± 5% in yogurts supplemented with whey protein were observed by González-Martínez et al. [58]. Less syneresis was observed during storage (6%–10%) for yogurts fermented with *L. delbrueckii* ssp. *bulgaricus*. In the study by Gustaw et al. [25], the 1% prebiotic addition caused a decrease in yogurt syneresis, what is in agreement with the results obtained in the present study. According to Aghajani et al. [26], the results of the syneresis measurements of probiotics samples showed significant increases (*p* < 0.05) over time. At the end of storage, the sample containing oligofructose showed the highest syneresis percentage, which was in line with our results. Regarding the addition of fructose (in the form of honey) to probiotic fermented dairy products, in the study by Mohan et al. [29], the unsweetened control samples exhibited extensive syneresis (*p* < 0.05) and behaved similarly to stirred yogurt samples, while all other samples were proper set-type yogurts.

Color is the first characteristic perceived by consumers, meaning it often influences consumer preferences, which is why it is considered one of the most important attributes in food products. For treatments in which oligofructose or fructose (white powder form) were used, color differences should not be observed. Castro et al. [22] found no differences (*p* > 0.05) among samples with the addition of different prebiotic agents in terms of their effects on the color attributes. Additionally, Mani-López et al. [44] reported that the color parameters L*, a*, and b* for yogurts and fermented milk products prepared with several mixtures of lactic acid bacteria were very similar and remained almost constant during storage.

In the present study, we found that the applied *Lactobacillus* starter cultures significantly affected the sensory properties of the prepared fermented milk samples. The use of heterofermented L. *brevis* B1 resulted in better sensory features in comparison to homofermented *L. johnsonii* K4. We also observed that oligofructose contributed positively to the sensory characteristics of the samples. The oligofructose properties mentioned by Roberfroid [59], including rounder mouthfeel and sustenance of flavor with reduced aftertaste, were probably responsible for the samples’ improved features. These phenomena were similar to those observed by Castro et al. [22]. In turn, Cardarelli et al. [60] observed significant differences between samples containing inulin, oligofructose, and oligosaccharides from honey (*p* < 0.05) during refrigerated storage of novel potentially synbiotic petit-Suisse cheese. The lowest acceptance was found for the control sample, while the highest individual mean acceptance was obtained for the sample with 10% oligofructose addition, which also provided the most consistent results (lowest standard deviation). According to Skryplonek [61], the evaluation showed that the tested yogurt-type beverages with added oligofructose had good sensory properties and the overall sensory quality exceeded 4 points on a 1–5-point scale. The quality of the beverages slightly decreased during the storage period, which was associated with the increased sour taste and decreased flavor intensity. In the study by Fornelli et al. [21], milk drinks containing *L. paracasei* and oligofructose showed the lowest levels of acceptability. The same results were obtained by Cruz et al. [20], who concluded that consumers would not choose yogurt products containing different concentrations of oligofructose, possibly due to changes in the product characteristics. These findings were in agreement with the subsequent results of the sensory assessment made by Glibowski and Zielińska [54]. Statistical analysis of the obtained scores showed that panelists found worse (*p* ≤ 0.05) levels of consistency for kefir products containing inulin and oligofructose. The flavor of kefir containing whole milk powder was better than oligofructose-supplemented kefirs. The same conclusion was deduced by Lee et al. [62], who evaluated the properties of fermented milk supplemented with *Cudrania tricuspidata*, the main component of which is fructose [63].

## 4. Materials and Methods

### 4.1. Strains and Culture Conditions

The research materials consisted of two strains of the *Lactobacillus* genus exhibiting probiotic properties: *Lactobacillus brevis* B1, isolated from bundz (a type of traditional Polish curd cheese made from sheep’s milk) (Polish patent no. p-426002); and *Lactobacillus johnsonii* K4, isolated from sauerkraut [52,64]. These strains were stored in frozen culture on de Man, Rogosa, and Sharpe (MRS, LabM, Bury, UK) media with 20% glycerol at −80 °C. Strains were cultivated on MRS broth at 37 °C for 24 h to a concentration of 10^9^ CFU/mL, then centrifuged (6000× *g*, 10 min) and washed in triplicate in sterile PBS (phosphate-buffered saline 1×, pH 7.4). Then, bacterial cells were resuspended in 100 mL of milk, obtaining an inoculum concentration of 10^7^ CFU/mL.

### 4.2. Preparation of Saccharides and Fermented Milk Samples 

Saccharides: Fructose (F) (Biofan, Piekary Śląskie, Poland) and oligofructose (O) (Raftilose P95, ORAFTI, Tienen, Belgium) were added as 1% and 2% (*w/v*) to the 100 mL portions of 3.2% fat UHT milk (Mlekovita, Wysokie Mazowieckie, Poland). Milk without saccharide addition was treated as the control sample. The milk samples were mixed until the F and OF were completely dissolved. Then, milk samples were inoculated with 10^7^ CFU of *L. brevis* B1 or *L. johnsonii* K4 culture and subjected to fermentation for 48 h at 37 °C. Each variant was prepared in triplicate. Samples were immediately cooled after incubation in an ice bath and then stored in a refrigerator (4 °C ± 1 °C) for 35 days. Table 5 summarizes the samples used in the experiment.

### 4.3. Microbiological Analysis

The measurements were performed during storage after 0, 7, 14, 21, 28, and 35 days according to ISO15214:2002. The cell counts were determined by serial dilution with sterile peptone water (LabM, Bury, UK) plated on MRS agar via the drop plate technique, then anaerobically incubated at 37 °C for 48 h. The results were expressed as log colony-forming units per 1 mL of the product (CFU/mL).

### 4.4. Acidity Analysis (pH)

The measurements were performed on days 0, 7, 14, 21, 28, and 35 using a pH meter (Elmetron CP 551, Zabrze, Poland). The results were read with an accuracy of 0.001. Before starting the measurements, the device was properly scaled. The test sample (20 g) was transferred to a clean measuring cell, the electrode was placed in the milk, then after the pH stabilized the result was read.

### 4.5. Texture Analysis

The firmness levels of fermented milk samples on days 0, 7, 21, and 35 of storage were analyzed using a TA.XT Plus Analyzer (Stable Micro Systems, Godalming, UK) with a 5 kg load cell at 20 °C, with a 2.5-cm-diameter cylindrical flat probe (P/25R). The measurement parameters were: speed, 1.0 mm/s; trigger force, 1 g; penetration depth, 5 mm. The experiment was conducted in five replications. The data were analyzed using Exponent v6.1.4.0 software (Stable Micro Mixtures, Surrey, UK).

The viscosity levels of the fermented milk samples on days 0, 7, 21, and 35 of storage (N/m^2^) were assessed using a Brookfield DV3T rotational viscometer (Brookfield Eng. Lab., Inc., Middleboro, MA, USA) at a constant shear rate (150 rpm/min), with a measurement temperature of 20 ± 1 °C (according to the manufacturer’s instructions). The analysis was carried out using an HA-02 spindle.

### 4.6. Color Measurement

The colors of fermented milk samples on days 0, 7, 21, and 35 of storage were measured with a Konica Minolta CM-2300d spectrophotometer (Konica Minolta Business Technologies, Inc., Osaka, Japan), following the method described by Buffa et al. [65]. The values for lightness (L*; 100  =  white, 0  =  black), redness (a*; +60  =  red; −60  =  green), and yellowness (b*; +60  =  yellow; −60  =  blue) were recorded. Three measurements were performed and the mean values were calculated.

The total color difference ΔE* was calculated using the following equation:

(1) $$\Delta E*=\sqrt{{\left(\Delta L*\right)}^{2}+{\left(\Delta a\right)}^{2}+{\left(\Delta b\right)}^{2}}$$

where ΔL*, Δa*, and Δb* represent the changes in the lightness, green–red coordinate, and blue–yellow coordinate in comparison to control samples, respectively.

### 4.7. Syneresis Measurement

The syneresis index values of fermented milk samples on days 0 and 35 of storage were determined according to the methodology proposed by Farnsworth et al. [66] with some modifications. Fermented milk samples (20 g) were prepared in centrifuge cups and centrifuged at 2500 rpm (average 640× *g*) for 10 min at 4 °C. The clear supernatant was collected and weighed, then syneresis values were calculated according to the following equation [67]:

Syneresis (%) = weight of supernatant (g)/weight of yogurt sample (g) × 100%(2)

### 4.8. Sensory Analysis

The quantitative descriptive profile (QDP) method was used following the procedure described in ISO standard 13299:2016 [68]. The panelists first individually chose the descriptors (attributes) of the appearance, odor, consistency, and flavor or taste of the samples. Next, the attributes were discussed, agreed upon, and defined (Supplementary material, Table S1). Five characteristics describing the odor of the samples (milky-fermentative, sterilization, sweet, irritating, other), one describing the appearance of the samples (color), three attributes describing the texture felt in the mouth (thickness, smoothness, viscosity), nine describing the taste and flavor (milky-fermentative, sterilization, sweet, sour, bitter, salty, flour, irritating and other), as well as other characteristics describing the overall quality were selected. The intensity of each attribute was measured by the panelists on a linear, unstructured 10-point scale (c.u.—contractual units), whereby 0 indicates low perception and 10 indicates high perception.

The sensory evaluation of the samples was performed by 10 trained panelists (experts) aged between 25 and 55, with good knowledge of all of the sensory methods, including profiling and fermented milk product analysis. The assessment was carried out in a room with controlled lighting, temperature, and humidity. Fermented milk samples were prepared in cylindrical containers (ø 50 mm, height 50 mm, volume 100 mL), covered with a lid, coded with 3-digit codes, placed in random order, then served at 7 °C to the evaluators. Each sample was analyzed in two independent replications, so that the mean values were based on 20 individual results, which were used for statistical analysis.

### 4.9. Statistical Analysis

The microbiological and physicochemical analyses, as well as the fermentation tests, were carried out in triplicate. Data from the microbiological, physicochemical, and instrumental analyses were subjected to two-way ANOVA. A Bonferroni correction was applied to all ANOVA results. Analysis of the sensory test results was performed using the Mann–Whitney U test. The results of the QDP were assessed based on the principal component analysis (PCA). Statistical significance was recognized when *p* < 0.05. All tests were performed using STATISTICA 13.3 PL software (StatSoft, Kraków, Poland).

## 5. Conclusions

This study was the first attempt to compare the influence of different sugar sources on the physicochemical, rheological, sensory, and microbiological quality of fermented milk products. In this study, it was found that oligofructose and fructose affect the physicochemical, rheological, sensory, and microbiological quality of fermented milk products. The addition of fructose in the amount of 1% caused an increase in sensory viscosity and the appearance of an irritating smell. The addition of oligofructose in the amount of 1% increased the hardness and caused the appearance of a sweet smell. The addition of oligofructose at up to 2% caused increased organoleptic viscosity and overall quality, as well as a more intensely sweet and sterile taste. Replacing fructose, which is often used in industry, with oligofructose appears to be beneficial from technological and nutritional perspectives.

The most important factor determining the quality of the samples was the strain of bacteria culture used for the fermentation process. Milk samples fermented with *L. brevis* B1 were characterized by better overall sensory quality than samples fermented with *L. johnsonii* K4. Both *L. brevis* B1 and *L. johnsonii* K4 exhibit probiotic properties (they are safe, have good survivability in model gastrointestinal tract conditions, exhibit antimicrobial properties, show adhesion to epithelial tissues, etc.), which was proven in previous studies [52,64]. According to these findings, independently of the bacteria strain added, all tested formulations were considered potentially probiotic products during their entire shelf life (35 days at 4 °C), with counts in the range of approximately 7–8 log CFU/mL. The most promising strain seems to be *L. brevis* B1.

It can be concluded that it is possible to develop potentially synbiotic products containing indigenous probiotic starter cultures of novel *Lactobacillus* strains isolated from Polish traditional fermented foods; however, it should be emphasized that this study is only the first step in the development of probiotic and synbiotic foods, since such foods must satisfy several criteria, including safety assessments in animal and human studies, before they can be called probiotic or synbiotic. Future studies will be focused on the optimization of the fermented milk production process, including detailed analysis of the acidification process, as well as analysis of the textural properties and compositions of the volatile compounds and the active flavor compounds in yogurt-like products.

## Figures and Tables

**Table 1:** Changes in pH values ± SD in fermented milk samples during storage.

Name of Sample	Time of Storage [Days]					
	0	7	14	21	28	35
K4	4.85 ± 0.17 ^a^	4.82 ± 0.15 ^a^	4.62 ± 0.17 ^bc^	4.39 ± 0.14 ^d^	4.32 ± 0.12 ^e^	4.28 ± 0.12 ^e^
K4 F1	4.70 ± 0.05 ^a^	4.68 ± 0.05 ^a^	4.50 ± 0.04 ^c^	4.24 ± 0.08 ^d^	4.20 ± 0.10 ^ef^	4.15 ± 0.11 ^ef^
K4 F2	4.83 ± 0.08 ^a^	4.80 ± 0.12 ^ab^	4.52 ± 0.03 ^c^	4.37 ± 0.28 ^e^	4.33 ± 0.30 ^ef^	4.26 ± 0.25 ^ef^
K4 O1	4.69 ± 0.01 ^a^	4.66 ± 0.02 ^b^	4.42 ± 0.07 ^d^	4.25 ± 0.05 ^ef^	4.22 ± 0.06 ^f^	4.21 ± 0.07 ^f^
K4 O2	4.68 ± 0.03 ^a^	4.66 ± 0.04 ^b^	4.41 ± 0.02 ^d^	4.20 ± 0.03 ^f^	4.22 ± 0.05 ^fg^	4.19 ± 0.06 ^g^
B1	4.38 ± 0.03 ^d^	4.37 ± 0.02 ^d^	4.33 ± 0.01 ^e^	4.21 ± 0.01 ^f^	4.21 ± 0.02 ^fg^	4.15 ± 0.02 ^g^
B1 F1	4.38 ± 0.12 ^d^	4.36 ± 0.11 ^de^	4.32 ± 0.10 ^e^	4.06 ± 0.11 ^fg^	4.06 ± 0.11 ^g^	4.02 ± 0.09 ^g^
B1 F2	4.31 ± 0.12 ^d^	4.36 ± 0.11 ^e^	4.32 ± 0.10 ^e^	4.10 ± 0.12 ^g^	4.12 ± 0.12 ^g^	4.11 ± 0.10 ^g^
B1 O1	4.31 ± 0.08 ^de^	4.32 ± 0.07 ^e^	4.32 ± 0.08 ^ef^	4.10 ± 0.09 ^g^	4.12 ± 0.08 ^g^	4.11 ± 0.07 ^g^
B1 O2	4.32 ± 0.08 ^de^	4.33 ± 0.06 ^e^	4.33 ± 0.05 ^ef^	4.10 ± 0.05 ^g^	4.11 ± 0.04 ^g^	4.13 ± 0.04 ^g^

Samples coded according to Materials and methods section. Values marked by different letters represent statistically significant differences between variables (storage time and strain) (*p* < 0.05); (*n* = 3).

**Table 2:** Changes in the viscosity values ± SD (mPa·s) for fermented milk samples during storage.

Name of Sample	Time of Storage [Days]			
	0	7	21	35
K4	39.56 ± 13.57 ^g^	61.81 ± 12.22 ^e^	45.45 ± 11.31 ^fg^	44.72 ± 5.37 ^fg^
K4 F1	42.39 ± 7.34 ^e^	70.12 ± 15.40 ^d^	51.72 ± 9.78 ^f^	48.67 ± 9.70 ^f^
K4 F2	39.33 ± 4.86 ^g^	69.59 ± 7.18 ^d^	58.25 ± 10.21 ^g^	52.76 ± 10.64 ^f^
K4 O1	40.45 ± 12.56 ^g^	88.77 ± 17.89 ^bc^	61.51 ± 16.41 ^g^	57.52 ± 13.27 ^e^
K4 O2	41.62 ± 2.65 ^g^	100.47 ± 7.95 ^b^	68.52 ± 8.55 ^e^	62.76 ± 5.44 ^g^
B1	64.62 ± 5.09 ^ed^	84.43 ± 18.19 ^c^	75.05 ± 0.64 ^cd^	55.38 ± 18.97 ^ef^
B1 F1	63.94 ± 10.57 ^e^	89.55 ± 9.36 ^bc^	79.89 ± 10.72 ^c^	60.75 ± 17.94 ^e^
B1 F2	61.61 ± 15.71 ^e^	94.80 ± 18.22 ^b^	87.04 ± 16.19 ^bc^	69.37 ± 13.59 ^d^
B1 O1	64.38 ± 18.59 ^ed^	114.15 ± 15.46 ^a^	90.90 ± 12.18 ^b^	78.71 ± 11.14 ^c^
B1 O2	73.20 ± 7.41 ^cd^	120.88 ± 19.46 ^a^	100.43 ± 12.87 ^b^	84.48 ± 15.29 ^c^

Samples coded according to Materials and methods section. Values marked by different letters represent statistically significant differences between variables (storage time and strain) (*p* < 0.05); (*n* = 3).

**Table 3:** Changes in firmness values ± SD (N) in fermented milk samples during storage.

Name of Sample	Time of Storage [Days]			
	0	7	21	35
K4	0.23 ± 0.02 ^b^	0.25 ± 0.05 ^a^	0.23 ± 0.05 ^b^	0.20 ± 0.00 ^c^
K4 F1	0.22 ± 0.01 ^bc^	0.24 ± 0.02 ^ab^	0.23 ± 0.02 ^b^	0.21 ± 0.01 ^c^
K4 F2	0.21 ± 0.01 ^c^	0.24 ± 0.01 ^ab^	0.22 ± 0.00 ^bc^	0.20 ± 0.01 ^c^
K4 O1	0.23 ± 0.00 ^b^	0.26 ± 0.01 ^a^	0.22 ± 0.00 ^c^	0.20 ± 0.01 ^c^
K4 O2	0.22 ± 0.01 ^bc^	0.24 ± 0.00 ^ab^	0.22 ± 0.00 ^bc^	0.20 ± 0.00 ^c^
B1	0.23 ± 0.04 ^b^	0.26 ± 0.01 ^a^	0.23 ± 0.03 ^ab^	0.22 ± 0.02 ^bc^
B1 F1	0.22 ± 0.00 ^bc^	0.24 ± 0.01 ^ab^	0.22 ± 0.01 ^bc^	0.21 ± 0.01 ^c^
B1 F2	0.22 ± 0.01 ^bc^	0.25 ± 0.02 ^a^	0.23 ± 0.00 ^bc^	0.21 ± 0.01 ^c^
B1 O1	0.23 ± 0.01 ^b^	0.25 ± 0.03 ^a^	0.23 ± 0.00 ^bc^	0.21 ± 0.00 ^c^
B1 O2	0.22 ± 0.01 ^bc^	0.26 ± 0.03 ^a^	0.23 ± 0.00 ^b^	0.23 ± 0.01 ^b^

Samples coded according to Materials and methods section. Values marked by different letters represent statistically significant differences between variables (storage time and strain) (*p* < 0.05); (*n* = 3).

**Table 4:** Changes in color values (L* a* b*) in fermented milk samples during storage.

Name of Sample	Time of Storage [Days]											
	0			7			21			35		
	L*	a*	b*	L*	a*	b*	L*	a*	b*	L*	a*	b*
K4	83.77 ± 3.20 ^a^	−1.34 ± 0.02 ^a^	7.52 ± 0.19 ^a^	90.01 ± 1.07 ^bc^	−1.47 ± 0.01 ^b^	7.76 ± 0.20 ^b^	92.21 ± 2.04 ^c^	−1.69 ± 0.07 ^c^	7.93 ± 0.07 ^bc^	84.65 ± 2.82 ^a^	−1.50 ± 0.06 ^b^	7.65 ± 0.32 ^ab^
K4 F1	83.06 ± 2.46 ^a^	−1.31 ± 0.03 ^a^	7.54 ± 0.24 ^a^	86.51 ± 3.05 ^ab^	−1.47 ± 0.12 ^b^	7.80 ± 0.27 ^b^	90.03 ± 3.04 ^bc^	−1.65 ± 0.15 ^c^	7.97 ± 0.47 ^c^	84.45 ± 2.10 ^a^	−1.45 ± 0.11^b^	7.71 ± 0.26 ^ab^
K4 F2	83.80 ± 2.44 ^a^	−1.36 ± 0.03 ^a^	7.54 ± 0.09 ^a^	85.22 ± 1.91 ^a^	−1.43 ± 0.07 ^b^	7.84 ± 0.16 ^b^	88.36 ± 1.62 ^b^	−1.67 ± 0.05 ^c^	7.97 ± 0.10 ^c^	83.65 ± 2.12 ^a^	−1.49 ± 0.06 ^b^	7.75 ± 0.05 ^b^
K4 O1	83.49 ± 1.94 ^a^	−1.33 ± 0.07 ^a^	7.53 ± 0.09 ^a^	89.49 ± 1.60 ^bc^	−1.51 ± 0.02 ^b^	7.89 ± 0.23 ^bc^	91.52 ± 1.75 ^c^	−1.69 ± 0.03 ^c^	8.02 ± 0.13 ^c^	85.50 ± 2.66 ^a^	−1.52 ± 0.02 ^b^	7.83 ± 0.45 ^b^
K4 O2	83.83 ± 4.10 ^a^	−1.32 ± 0.02 ^a^	7.59 ± 0.022 ^a^	88.20 ± 2.21 ^b^	−1.39 ± 0.03 ^b^	7.94 ± 0.17 ^bc^	90.99 ± 0.56 ^c^	−1.65 ± 0.02 ^c^	8.09 ± 0.41 ^c^	84.67 ± 0.50 ^a^	−1.55 ± 0.01 ^bc^	7.90 ± 0.05 ^bc^
B1	85.33 ± 2.47 ^a^	−1.31 ± 0.04 ^a^	7.53 ± 0.009 ^a^	89.02 ± 2.67 ^b^	−1.41 ± 0.05 ^ab^	7.65 ± 0.27 ^ab^	92.48 ± 4.23 ^c^	−1.65 ± 0.07 ^c^	7.82 ± 0.15 ^b^	84.85 ± 1.99 ^a^	−1.52 ± 0.07 ^b^	7.70 ± 0.02 ^ab^
B1 F1	85.49 ± 3.04^a^	−1.27 ± 0.05 ^a^	7.56 ± 0.23 ^a^	86.63 ± 2.30 ^ab^	−1.38 ± 0.04 ^ab^	7.70 ± 0.21^ab^	88.26 ± 1.78 ^b^	−1.60 ± 0.16 ^c^	7.86 ± 0.40 ^b^	84.06 ± 0.27 ^a^	−1.43 ± 0.03 ^b^	7.80 ± 0.10 ^b^
B1 F2	85.36 ± 1.86 ^a^	−1.31 ± 0.02 ^a^	7.57 ± 0.16 ^a^	86.47 ± 2.68 ^ab^	−1.44 ± 0.13 ^b^	7.80 ± 0.07 ^b^	87.70 ± 1.48 ^b^	−1.57 ± 0.09 ^bc^	7.87 ± 0.97 ^b^	83.87 ± 0.57 ^a^	−1.53 ± 0.12 ^bc^	7.84 ± 0.26 ^b^
B1 O1	85.17 ± 3.81 ^a^	−1.26 ± 0.08 ^a^	7.53 ± 0.17 ^a^	88.67 ± 3.15 ^b^	−1.45 ± 0.03 ^b^	7.89 ± 0.34 ^b^	90.95 ± 0.26 ^c^	−1.70 ± 0.03 ^c^	7.95 ± 0.14 ^c^	84.50 ± 1.59 ^a^	−1.49 ± 0.08 ^b^	7.87 ± 0.13 ^bc^
B1 O2	85.04 ± 6.55 ^a^	−1.26 ± 0.10 ^a^	7.58 ± 0.45 ^a^	87.26 ± 3.08 ^b^	−1.40 ± 0.06 ^ab^	7.95 ± 0.28 ^c^	90.48 ± 2.59 ^c^	−1.64 ± 0.12 ^c^	8.03 ± 0.08 ^c^	83.72 ± 0.48 ^a^	−1.45 ± 0.03 ^b^	7.97 ± 0.18 ^c^

Samples coded according to Materials and methods section. Values denoted by different letters represent statistically significant differences between variables (storage time and strains) (*p* < 0.05); (*n* = 3).

**Table 5:** Experimental design.

Strain Inoculation	Saccharide Addition				
	Control	Fructose (F)		Oligofructose (O)	
	0%	1%	2%	1%	2%
*L. brevis* B1	B1	B1 F1	B1 F2	B1 O1	B1 O2
*L. johnsonii* K4	K4	K4 F1	K4 F2	K4 O1	K4 O2

## Data Availability

Data will be provided upon request.

## Supplementary Materials

The following are available online: Table S1: Sensory attributes used for yogurt profiling, their definitions, and their intensity range.

## Sample Availability

Samples of the compounds are available from the authors.
