# Supplementary material for: Effects of Fructose and Oligofructose Addition on Milk Fermentation Using Novel Lactobacillus Cultures to Obtain High-Quality Yogurt-like Products
Source: Molecules. 2021 Sep 22;26(19):5730. doi: 10.3390/molecules26195730 (PMC8510434; doi:10.3390/molecules26195730)
Supplement: Supplementary file 1 [file molecules-26-05730-s001.zip › molecules-1353306-supplementary.pdf]

Supplementary Materials

Table S1. Sensory attribute for fermented milk samples profiling, their definitions and intensity range.

Attributes	Definitions	Intensity range
Milky-fermentative smell	Mild smell characteristic of lactic fermentation of products such as yoghurt, sour milk	none - very intensive
Sterilization smell	The smell characteristic of boiled milk	none - very intensive
Sweet smell	Basic odour quality - no definition required	none - very intensive
Irritating smell	smell with an unpleasant, irritating sensation when the sample is spread in the mouth	none - very intensive
Other smell	smell not matching the above-mentioned	none - very intensive
Color tone	Colour intensity of the product	light white - dark cream
Density	Perception of the product's density in the mouth	thin - thick
Smoothness	Degree of smoothness in the product's surface (grainy/ smooth appearance)	rough - smooth
Viscosity	Feeling of sticking to the palate, ductility	low-high
Milky-fermentative taste	Mild taste characteristic of lactic fermentation of products such as yoghurt, sour milk	none - very intensive
Sterilization taste	The taste characteristic of boiled milk	none - very intensive
Sour taste	Basic taste quality - no definition required	none - very intensive
Sweet taste	Basic taste quality - no definition required	none - very intensive
Salty taste	Basic taste quality - no definition required	none - very intensive
Bitter taste	Basic taste quality - no definition required	none - very intensive
Floury taste	Characteristic for the taste of flour dissolved in water	none - very intensive
Irritating smell	Taste with an unpleasant, irritating sensation when the sample is spread in the mouth	none - very intensive
Other smell	Taste not matching the above-mentioned	none - very intensive
Overall quality	Overall impression of the harmony of positive attributes, with no or only slight negative notes	low-high
